# Supplementary material for: HIVprotI: an integrated web based platform for prediction and design of HIV proteins inhibitors
Source: J Cheminform. 2018 Mar 9;10:12. doi: 10.1186/s13321-018-0266-y (PMC5845081; doi:10.1186/s13321-018-0266-y)
Supplement: Supplementary file 2 — Additional file 2. Source code of HIVProtI web server. [file 13321_2018_266_MOESM2_ESM.zip › HIVprotI_Source-code/hivprotI/weka-3-6-6/doc/weka/gui/boundaryvisualizer/BoundaryVisualizer.html]

BoundaryVisualizer


---


|  |  |  |
| --- | --- | --- |
| **Overview** \| **Package** \| **Class** \| **Tree** \| **Deprecated** \| **Index** \| **Help** \| **Weka's home** | |  |
| **PREV CLASS**   **NEXT CLASS** | **FRAMES**    **NO FRAMES**     **All Classes** |  |
| SUMMARY: NESTED \| FIELD \| CONSTR \| METHOD | DETAIL: FIELD \| CONSTR \| METHOD |  |


---


## weka.gui.boundaryvisualizer Class BoundaryVisualizer

```
java.lang.Object
  java.awt.Component
      java.awt.Container
          javax.swing.JComponent
              javax.swing.JPanel
                  weka.gui.boundaryvisualizer.BoundaryVisualizer
```

**All Implemented Interfaces:**: java.awt.image.ImageObserver, java.awt.MenuContainer, java.io.Serializable, javax.accessibility.Accessible, TechnicalInformationHandler

---

``` public class BoundaryVisualizer extends javax.swing.JPanel implements TechnicalInformationHandler ```

BoundaryVisualizer. Allows the visualization of classifier decision
boundaries in two dimensions. A supplied classifier is first
trained on supplied training data, then a data generator (currently
using kernels) is used to generate new instances at points fixed in
the two visualization dimensions but random in the other
dimensions. These instances are classified by the classifier and
plotted as points with colour corresponding to the probability
distribution predicted by the classifier. At present, 2 \* 2^(#
non-fixed dimensions) points are generated from each kernel per
pixel in the display. In practice, fewer points than this are
actually classified because kernels are weighted (on a per-pixel
basis) according to the fixexd dimensions and kernels corresponding
to the lowest 1% of the weight mass are discarded. Predicted
probability distributions are weighted (acording to the fixed
visualization dimensions) and averaged to produce an RGB value for
the pixel. For more information, see

Eibe Frank and Mark Hall (2003). Visualizing Class Probability
Estimators. Working Paper 02/03, Department of Computer Science,
University of Waikato.

**Since:**
:   1.0

**Version:**
:   $Revision: 7381 $

**Author:**
:   Mark Hall

**See Also:**: `JPanel`, Serialized Form

---

**Nested Class Summary**

**Nested classes/interfaces inherited from class javax.swing.JComponent**

`javax.swing.JComponent.AccessibleJComponent`

**Nested classes/interfaces inherited from class java.awt.Component**

`java.awt.Component.BaselineResizeBehavior`


**Field Summary**

**Fields inherited from class javax.swing.JComponent**

`TOOL_TIP_TEXT_KEY, UNDEFINED_CONDITION, WHEN_ANCESTOR_OF_FOCUSED_COMPONENT, WHEN_FOCUSED, WHEN_IN_FOCUSED_WINDOW`

**Fields inherited from class java.awt.Component**

`BOTTOM_ALIGNMENT, CENTER_ALIGNMENT, LEFT_ALIGNMENT, RIGHT_ALIGNMENT, TOP_ALIGNMENT`

**Fields inherited from interface java.awt.image.ImageObserver**

`ABORT, ALLBITS, ERROR, FRAMEBITS, HEIGHT, PROPERTIES, SOMEBITS, WIDTH`


**Constructor Summary**

`BoundaryVisualizer()`
  
          Creates a new `BoundaryVisualizer` instance.


| **Method Summary** | |
| --- | --- |
| `static void` | `createNewVisualizerWindow(Classifier classifier, Instances instances)`             Creates a new GUI window with all of the BoundaryVisualizer trappings, |
| `static boolean` | `getExitIfNoWindowsOpen()`             Gets whether System.exit gets called after the last window gets closed |
| `Instances` | `getInstances()`             Get the training instances |
| `TechnicalInformation` | `getTechnicalInformation()`             Returns an instance of a TechnicalInformation object, containing detailed information about the technical background of this class, e.g., paper reference or book this class is based on. |
| `java.lang.String` | `globalInfo()`             Returns a string describing this tool |
| `static void` | `main(java.lang.String[] args)`             Main method for testing this class |
| `void` | `plotTrainingData()`             Plots the training data on-screen. |
| `void` | `setClassifier(Classifier newClassifier)`             Set a classifier to use |
| `static void` | `setExitIfNoWindowsOpen(boolean value)`             Sets whether System.exit gets called when no more windows are open. |
| `void` | `setInstances(Instances inst)`             Set the training instances |
| `void` | `setInstancesFromFileQ()`             Queries the user for a file to load instances from, then loads the instances in a background process. |
| `int` | `setUpBoundaryPanel()`             Sets up the BoundaryPanel object so that it is ready for plotting. |
| `void` | `stopPlotting()`             Stops the plotting thread. |

**Methods inherited from class javax.swing.JPanel**

`getAccessibleContext, getUI, getUIClassID, setUI, updateUI`

**Methods inherited from class javax.swing.JComponent**

`addAncestorListener, addNotify, addVetoableChangeListener, computeVisibleRect, contains, createToolTip, disable, enable, firePropertyChange, firePropertyChange, firePropertyChange, getActionForKeyStroke, getActionMap, getAlignmentX, getAlignmentY, getAncestorListeners, getAutoscrolls, getBaseline, getBaselineResizeBehavior, getBorder, getBounds, getClientProperty, getComponentPopupMenu, getConditionForKeyStroke, getDebugGraphicsOptions, getDefaultLocale, getFontMetrics, getGraphics, getHeight, getInheritsPopupMenu, getInputMap, getInputMap, getInputVerifier, getInsets, getInsets, getListeners, getLocation, getMaximumSize, getMinimumSize, getNextFocusableComponent, getPopupLocation, getPreferredSize, getRegisteredKeyStrokes, getRootPane, getSize, getToolTipLocation, getToolTipText, getToolTipText, getTopLevelAncestor, getTransferHandler, getVerifyInputWhenFocusTarget, getVetoableChangeListeners, getVisibleRect, getWidth, getX, getY, grabFocus, isDoubleBuffered, isLightweightComponent, isManagingFocus, isOpaque, isOptimizedDrawingEnabled, isPaintingForPrint, isPaintingTile, isRequestFocusEnabled, isValidateRoot, paint, paintImmediately, paintImmediately, print, printAll, putClientProperty, registerKeyboardAction, registerKeyboardAction, removeAncestorListener, removeNotify, removeVetoableChangeListener, repaint, repaint, requestDefaultFocus, requestFocus, requestFocus, requestFocusInWindow, resetKeyboardActions, reshape, revalidate, scrollRectToVisible, setActionMap, setAlignmentX, setAlignmentY, setAutoscrolls, setBackground, setBorder, setComponentPopupMenu, setDebugGraphicsOptions, setDefaultLocale, setDoubleBuffered, setEnabled, setFocusTraversalKeys, setFont, setForeground, setInheritsPopupMenu, setInputMap, setInputVerifier, setMaximumSize, setMinimumSize, setNextFocusableComponent, setOpaque, setPreferredSize, setRequestFocusEnabled, setToolTipText, setTransferHandler, setVerifyInputWhenFocusTarget, setVisible, unregisterKeyboardAction, update`

**Methods inherited from class java.awt.Container**

`add, add, add, add, add, addContainerListener, addPropertyChangeListener, addPropertyChangeListener, applyComponentOrientation, areFocusTraversalKeysSet, countComponents, deliverEvent, doLayout, findComponentAt, findComponentAt, getComponent, getComponentAt, getComponentAt, getComponentCount, getComponents, getComponentZOrder, getContainerListeners, getFocusTraversalKeys, getFocusTraversalPolicy, getLayout, getMousePosition, insets, invalidate, isAncestorOf, isFocusCycleRoot, isFocusCycleRoot, isFocusTraversalPolicyProvider, isFocusTraversalPolicySet, layout, list, list, locate, minimumSize, paintComponents, preferredSize, printComponents, remove, remove, removeAll, removeContainerListener, setComponentZOrder, setFocusCycleRoot, setFocusTraversalPolicy, setFocusTraversalPolicyProvider, setLayout, transferFocusBackward, transferFocusDownCycle, validate`

**Methods inherited from class java.awt.Component**

`action, add, addComponentListener, addFocusListener, addHierarchyBoundsListener, addHierarchyListener, addInputMethodListener, addKeyListener, addMouseListener, addMouseMotionListener, addMouseWheelListener, bounds, checkImage, checkImage, contains, createImage, createImage, createVolatileImage, createVolatileImage, dispatchEvent, enable, enableInputMethods, firePropertyChange, firePropertyChange, firePropertyChange, firePropertyChange, firePropertyChange, getBackground, getBounds, getColorModel, getComponentListeners, getComponentOrientation, getCursor, getDropTarget, getFocusCycleRootAncestor, getFocusListeners, getFocusTraversalKeysEnabled, getFont, getForeground, getGraphicsConfiguration, getHierarchyBoundsListeners, getHierarchyListeners, getIgnoreRepaint, getInputContext, getInputMethodListeners, getInputMethodRequests, getKeyListeners, getLocale, getLocation, getLocationOnScreen, getMouseListeners, getMouseMotionListeners, getMousePosition, getMouseWheelListeners, getName, getParent, getPeer, getPropertyChangeListeners, getPropertyChangeListeners, getSize, getToolkit, getTreeLock, gotFocus, handleEvent, hasFocus, hide, imageUpdate, inside, isBackgroundSet, isCursorSet, isDisplayable, isEnabled, isFocusable, isFocusOwner, isFocusTraversable, isFontSet, isForegroundSet, isLightweight, isMaximumSizeSet, isMinimumSizeSet, isPreferredSizeSet, isShowing, isValid, isVisible, keyDown, keyUp, list, list, list, location, lostFocus, mouseDown, mouseDrag, mouseEnter, mouseExit, mouseMove, mouseUp, move, nextFocus, paintAll, postEvent, prepareImage, prepareImage, remove, removeComponentListener, removeFocusListener, removeHierarchyBoundsListener, removeHierarchyListener, removeInputMethodListener, removeKeyListener, removeMouseListener, removeMouseMotionListener, removeMouseWheelListener, removePropertyChangeListener, removePropertyChangeListener, repaint, repaint, repaint, resize, resize, setBounds, setBounds, setComponentOrientation, setCursor, setDropTarget, setFocusable, setFocusTraversalKeysEnabled, setIgnoreRepaint, setLocale, setLocation, setLocation, setName, setSize, setSize, show, show, size, toString, transferFocus, transferFocusUpCycle`

**Methods inherited from class java.lang.Object**

`equals, getClass, hashCode, notify, notifyAll, wait, wait, wait`

**Constructor Detail**

### BoundaryVisualizer

```
public BoundaryVisualizer()
```

:   Creates a new `BoundaryVisualizer` instance.


**Method Detail**

### globalInfo

```
public java.lang.String globalInfo()
```

:   Returns a string describing this tool

    :   **Returns:**: a description of the tool suitable for displaying in various Weka GUIs

---


### getTechnicalInformation

```
public TechnicalInformation getTechnicalInformation()
```

:   Returns an instance of a TechnicalInformation object, containing
    detailed information about the technical background of this class,
    e.g., paper reference or book this class is based on.

    :   **Specified by:**: `getTechnicalInformation` in interface `TechnicalInformationHandler`
    :   **Returns:**: the technical information about this class

---


### setClassifier

```
public void setClassifier(Classifier newClassifier)
                   throws java.lang.Exception
```

:   Set a classifier to use

    :   **Parameters:**: `newClassifier` - the classifier to use **Throws:**: `java.lang.Exception` - if an error occurs

---


### getInstances

```
public Instances getInstances()
```

:   Get the training instances

    :   **Returns:**: the training instances

---


### setInstances

```
public void setInstances(Instances inst)
                  throws java.lang.Exception
```

:   Set the training instances

    :   **Parameters:**: `inst` - the instances to use **Throws:**: `java.lang.Exception`

---


### setInstancesFromFileQ

```
public void setInstancesFromFileQ()
```

:   Queries the user for a file to load instances from, then loads the
    instances in a background process. This is done in the IO
    thread, and an error message is popped up if the IO thread is busy.

---


### setUpBoundaryPanel

```
public int setUpBoundaryPanel()
                       throws java.lang.Exception
```

:   Sets up the BoundaryPanel object so that it is ready for plotting.

    :   **Returns:**: an error code:

---


### plotTrainingData

```
public void plotTrainingData()
                      throws java.lang.Exception
```

:   Plots the training data on-screen. Also does all of the setup required
    for this to work.

    :   **Throws:**: `java.lang.Exception`

---


### stopPlotting

```
public void stopPlotting()
```

:   Stops the plotting thread.

---


### setExitIfNoWindowsOpen

```
public static void setExitIfNoWindowsOpen(boolean value)
```

:   Sets whether System.exit gets called when no more windows are open.

    :   **Parameters:**: `value` - if TRUE then a System.exit call is ossued after the last window gets closed.

---


### getExitIfNoWindowsOpen

```
public static boolean getExitIfNoWindowsOpen()
```

:   Gets whether System.exit gets called after the last window gets closed

    :   **Returns:**: TRUE if System.exit gets called after last window got closed.

---


### createNewVisualizerWindow

```
public static void createNewVisualizerWindow(Classifier classifier,
                                             Instances instances)
                                      throws java.lang.Exception
```

:   Creates a new GUI window with all of the BoundaryVisualizer trappings,

    :   **Parameters:**: `classifier` - The classifier to use in the new window. May be null.: `instances` - The dataset to visualize on in the new window. May be null. **Throws:**: `java.lang.Exception`

---


### main

```
public static void main(java.lang.String[] args)
```

:   Main method for testing this class

    :   **Parameters:**: `args` - a `String[]` value


---


|  |  |  |
| --- | --- | --- |
| **Overview** \| **Package** \| **Class** \| **Tree** \| **Deprecated** \| **Index** \| **Help** \| **Weka's home** | |  |
| **PREV CLASS**   **NEXT CLASS** | **FRAMES**    **NO FRAMES**     **All Classes** |  |
| SUMMARY: NESTED \| FIELD \| CONSTR \| METHOD | DETAIL: FIELD \| CONSTR \| METHOD |  |


---
